# Outcomes of combined hip procedure with dual mobility cup versus osteosynthesis for acetabular fractures in elderly patients: a retrospective observational cohort study of fifty one patients

**DOI:** 10.1007/s00264-020-04757-w

**Published:** 2020-08-09

**Authors:** Xavier Lannes, Kevin Moerenhout, Hong Phuoc Duong, Olivier Borens, Sylvain Steinmetz

**Affiliations:** 1 Department of Orthopaedic Surgery and Traumatology, Centre Hospitalier Universitaire Vaudois (CHUV), Rue du Bugnon 46, 1011 Lausanne, Switzerland; 2 Institute for Research in Rehabilitation, Clinique romande de réadaptation Sion, Avenue du Grand-Champsec 90, 1950 Sion, Switzerland

**Keywords:** Combined hip procedure, Open reduction internal fixation, Dual mobility cup, Acetabular fractures, Center of rotation, Elderly patient

## Abstract

**Purposes:**

Acetabular fractures are more and more common in the elderly. Open reduction and internal fixation (ORIF) may lead to poor outcomes and high revision rates. Primary total hip arthroplasty (THA) combined with internal fixation, also known as the combined hip procedure (CHP), associated with dual mobility cup (DM-CHP) could be an efficient procedure in selected elderly patients. The aim of this study is to compare functional and radiological outcomes between ORIF and DM-CHP.

**Methods:**

Between 2007 and 2018, 51 patients older than 65 years were surgically treated for acetabular fractures. Twenty-six patients were treated by DM-CHP and 25 by ORIF. Each group was divided into two subgroups regarding a single or combined approach. Hospital stay, surgical time, intraoperative blood loss, and complications were documented. The Harris Hip Score (HHS) was used for measuring the functional outcome. Radiological analysis was used to assess the centre of rotation in the DM-CHP group.

**Results:**

Median surgery time and intra-operative blood loss were higher in DM-CHP than those in ORIF. Early medical complication rate was higher for a combined approach as compared with a single posterior approach in DM-CHP (*p* = 0.003). Dislocation rate was 7.7% in DM-CHP. Revision rate was higher in ORIF (20% versus 7.7%). HHS was similar in both groups.

**Conclusions:**

DM-CHP leads to similar functional outcomes and less revision than ORIF. This study strengthens the practice of using only the posterior approach for primary THA in the elderly. Dual mobility is a valid therapeutic option for acetabular fractures in elderly patients.

## Introduction

Acetabular fractures in patients aged 60 years and older have steadily become more frequent with a report of a 2.4-fold increase in incidence [1]. Kannus et al. [2] have also shown a rise of 23% of osteoporotic pelvic fractures with an ever older population. Low-energy trauma (LE) accounts for more than 50% of elderly acetabular fractures [3], which normally lead to a “senior fracture pattern”. This pattern consists of a displaced anterior column fracture associated with a posterior column fragment with a large portion of the quadrilateral surface displaced medially and cranially [1].

Some studies have shown that there are predictable factors of poor outcome concerning open reduction and internal fixation (ORIF), namely, age and non-anatomical reduction [4], superior anteromedial dome impaction [5], involvement of the posterior wall with marginal impaction or comminution [6], and femoral head damage [7]. About 17% of the patients [8] will need secondary total hip arthroplasty (THA) after acetabular fracture treated by ORIF. This surgery can be technically difficult and lead to high complication rates [9].

ORIF associated with primary THA, also known as the “ combined hip procedure” (CHP), seems to be an attractive procedure with satisfactory results in selected patients [10–13] and lead to equivalent non-fatal complication rate as ORIF [8]. Compared with ORIF, there is no need for an anatomic reduction, but there must be an adequate stability and bone stock to achieve THA. This surgical procedure allows for early mobilization with total weight-bearing [12], which should theoretically lead to fewer post-operative complications [14].

Dislocation rate in acetabular fractures treated by acute THA can run up to 23% [11, 15]. Dual mobility cups (DMC) are associated with lower rates of dislocation in primary surgery [16], revision surgery [17], and in primary THA for femoral neck fractures [18]. DMC could therefore be a useful tool for acetabular fractures.

Our hypothesis is that dual mobility-combined hip procedure (DM-CHP) is a safe procedure, with comparable clinical results and less early revision rate than ORIF in selected patients older than 65 years treated for acetabular fractures.

The aim of this study is to compare the functional and radiological outcomes between DM-CHP versus ORIF in consecutive patients older than 65 years treated for acetabular fractures.

## Patients and methods

### Patients

We retrospectively reviewed in our level I trauma department consecutively patients older than 65 years with acetabular fracture on a native hip who were treated surgically with ORIF or DM-CHP from January 2007 to September 2018.

Patients were divided into two groups based on two surgical methods: ORIF and DM-CHP. The indications for DM-CHP were severe comminuted articular fractures and marginal impaction (especially in weight-bearing area), fracture displacement of more than 20 mm, concomitant femoral head or neck fracture, marked osteoporosis (Singh index ≤ 2), and pre-existing symptomatic hip degenerative joint disease. ORIF group was divided into two subgroups: operated by a single approach (SA-ORIF) (anterior or posterior) and operated by a combined approach (CA-ORIF) (anterior and posterior). DM-CHP was subdivided in a single approach (SA-CHP) and combined approach (CA-CHP).

### Surgical procedure

Surgery was done by two of our senior lower limb trauma surgeons. For the ORIF group, the surgical approach was decided depending on the fracture pattern [19]. When the anterior column was involved, a modified Stoppa approach was realized. For posterior column or posterior wall fractures, the Kocher-Langenbeck approach was performed. Finally, in both column fractures, anterior followed by posterior approaches were used. Osteosynthesis was realized with a suprapectineal quadrilateral buttress plate (PRO Quadrilateral surface plates, Stryker®, Kalamazoo, MC, USA) when anterior column was involved and by a reconstruction plate (Matta pelvic plates, Stryker®, Kalamazoo, MC, USA) or one-third tubular plate (DePuySynthes®, New Brunswick, NJ, USA) when posterior column or wall was involved. For four patients, the outer window of the ilioinguinal approach was used in combination with a modified Stoppa approach.

In the DM-CHP group, osteosynthesis was done first, followed by the THA. When the anterior column was involved, a modified Stoppa approach was realized, and osteosynthesis was done with a suprapectineal quadrilateral buttress plate. Then, the patient was positioned in lateral decubitus, and a Kocher-Langenbeck approach was used for posterior column or wall osteosynthesis followed by the THA. When the anterior column was not involved, Kocher-Langenbeck approach was performed with posterior column or wall osteosynthesis followed by THA. Arthroplasty involved an acetabular reinforcement ring (Ganz ring, Zimmer-Biomet®, Warsaw, Indiana, USA) with a cemented dual mobility cup (Symbol cup DM, Dedienne santé®, Mauguio, France). Various stems were used throughout the study period (Symbios®, Yverdon-les-Bains, Switzerland). Stems were cemented or uncemented regarding the proximal femur bone quality.

### Clinical and radiological assessment

Characteristics of patients included age, gender, American Society of Anesthesiologists score (ASA), Charlson comorbidity index (CCI) [20], fracture patterns, Singh index, energy of trauma, surgery time, intra-operative blood loss, postoperative length of stay, early medical complications, surgical site infection, dislocation rate, late complications, Harris hip score (HHS), surgical revision, analysis of post-operative radiographs, and computed tomography (CT).

Follow-up time was calculated from the day of surgery to the date of the most recent visit or when the patient died. Charlson comorbidity index were divide into 3 groups according to the scores: 0, 1–3, and > 4 [20]. The surgical approach, surgery time (incision to end of wound closure), and intra-operative blood loss were retrospectively obtained from the surgical and anaesthesiologic reports. Post-operative length of stay (defined as the number of calendar days from the operation to hospital discharge), early medical complications (from the surgery day to 6 weeks), surgical site infection, dislocations, osteoarthritis, surgery revision, heterotopic ossification (HO), and post-operative functional outcome score (Harris Hip Score) were retrospectively reviewed from the medical reports.

Fracture patterns were classified by two fellowship trained senior surgeons according to the Letournel classification [19] and the AO classification (Arbeitsgemeinschaft für Osteosynthesefragen) on pre-operative X-rays and CT scan. The Singh index was evaluated on pre-operative anteroposterior pelvic X-rays. The lower index of both femurs was taken as reference. For the ORIF group, the quality of postoperative fracture reduction was analyzed by CT scan examining the largest remaining gap [4]. The reduction quality was expressed in millimeters (mm). A gap between fracture fragments of ≤ 2 mm was considered as anatomic.

Anteroposterior pelvic, obturator, and iliac view X-rays for ORIF were assessed at every clinical follow-up searching for osteoarthritis, osteonecrosis, loss of reduction, and HO. Concerning DM-CHP, radiologic follow-up was assessed by anteroposterior pelvic X-ray and axial X-ray of the operated hip searching for loosening and HO. The degree of HO was evaluated using the Brooker classification [21]. The biomechanical centre of rotation (COR) reconstruction was calculated as the difference from the contralateral hip [10] with TraumaCad® software (Brainlab®, Munich, Germany).

### Statistical analysis

Continuous variables with normal distribution (age, HHS) were presented as mean and standard deviation. Continuous variables with non-normal distribution (intra-operative blood loss, surgery time, length of post-operative stay) were presented as median values and range. Categorical variables were presented as proportions. The Fischer exact test was used for hypothesis testing of binary variables, and the Mann Whitney *U* test or Student *t* test was used for continuous variables. Binary logistic regression was used to assess the correlation between CCI and early complications rate in the two groups (odds ratio with the 95% confidence interval (CI). The relationship between HHS and COR reconstruction was assessed by the Pearson correlation coefficient. All analyses were performed using STATA 15.0 soft-ware (StataCorp, College Station, TX, USA), and a *P* value of < 0.05 was considered significant.

## Results

### DM-CHP versus ORIF

Fifty-one patients met the inclusion criteria, from whom 25 patients were treated with ORIF and 26 with DM-CHP. Characteristics of both groups, including age, CCI, ASA score, and fracture patterns were similar. Gender distribution showed that the frequency of men was 80% (20/25) in the ORIF group compared with 58% (15/26) in the DM-CHP group (*p* = 0.09, Table 1).

**Table 1:** Characteristics of two groups patients

Characteristics	ORIF	DM-CHP	*P* value
	*N* = 25	*N* = 26	
Age (years), mean ± SD (range)	75 ± 8 (range 66–92)	78 ± 6 (range 66–88)	0.16
Gender, *n* (%)			0.09
Women	5 (20%)	11 (42%)	
Men	20 (80%)	15 (58%)	
Charlson comorbidity index, *n* (%)			0.89
0	8 (32%)	10 (38%)	
1–3	13 (52%)	12 (46%)	
≥ 4	4 (16%)	4 (15%)	
ASA score, *n* (%)			0.35
1	1 (4%)	0	
2	12 (48%)	14 (54%)	
3	12(48%)	10 (38%)	
4	0	2 (8%)	
Letournel classification ^1^, *n* (%)			0.13
Posterior wall	5 (20%)	3 (11.5%)	
Posterior column	1 (4%)	0	
Anterior wall	1 (4%)	0	
Anterior column	0	1 (3.9%)	
Transverse	0	5 (19.2%)	
Posterior column, posterior wall	0	1 (3.9%)	
Transverse, posterior wall	0	2 (7.7%)	
T-type	2 (8%)	3 (11.5%)	
AC-PHT	9 (36%)	6 (23.1%)	
Both columns	7 (28%)	5 (19.2%)	
AO classification, *n* (%)			0.56
A1-A3	7 (28%)	5 (19%)	
B1-B3	11 (44%)	16 (62%)	
C	7 (28%)	5 (19%)	
Singh index (*n*), median (range)	3 (2–5)	2 (1–4)	
Energy of trauma			
Low Energy (LE), *n* (%)	14 (56%)	17 (65.4%)	
High Energy (HE), *n* (%)	11 (44%)	9 (34.6%)	
Surgical approach, *n* (%)			
Anterior (SA)	13 (52%)	0	
Posterior (SA)	8 (32%)	18 (69%)	
Anterior + posterior (CA)	4 (16%)	8 (31%)	
Intraoperative blood loss (ml), median (range)	500 (200–1800)	1000 (369–1700)	0.006
Surgery time (minutes), median (range)	125 (54–305)	185 (106–272)	< 0.001
Length of post-operative stay	14 (1–46)	11 (3–46)	0.32
(days), median (range)			
Early medical complications, *n* (%)	8 (32%)	8 (31%)	0.94
Surgical site infection, *n* (%)	1 (4%)	2 (7.7%)	0.57
Revision, *n* (%)	5 (20%)	2 (7,7%)	0.57
Harris Hip Score, mean ± SD	68.25 ± 21.20	72.36 ± 11.65	0.47
Follow-up (months), median (range)	12 (range 1–56)	12 (range 1–96)	0.1

*ORIF* open reduction internal fixation, *DM-CHP* dual mobility-combined hip procedure

*AO* Arbeitsgemeinschaft für Osteosynthesefragen, *SA* single approach, *CA* combined approach

*ASA* American Society of Anesthesiologists, *AC-PHT* anterior column posterior hemi transverse

^1^Fracture patterns according to Letournel classification [19]

Surgical approaches are reported in Table 1. Median surgery time (*p* < 0.001) and intra-operative blood loss (*p* = 0.006) were higher in the DM-CHP group. The length of post-operative stay, HHS, early complication rate, and infection rate were similar (Table 1).

Early complications are summarized in Table 2. We recorded one death due to cardiac arrest following a Mendelson syndrome in the ORIF group during hospital stay and another one from unrelated cause after hospital stay in DM-CHP.

**Table 2:** Comparison between single and combined approaches in ORIF and DM-CHP group

		ORIF			DM-CHP	
	SA-ORIF	CA-ORIF	P value	SA-CHP	CA-CHP	*P* value
	*n* = 21	*n* = 4		*n* = 18	*n* = 8	
Intra-operative blood loss (ml), median	500	1000	0.16	800	1000	0.04
(ml), (range)	(200–1800)	(250–1750)		(369–1100)	(400–1700)	
Surgery time (minutes), median	110	145	0.06	168	212.5	0.04
(minutes), (range)	(54–305)	(107–246)		(106–272)	(165–250)	
Early medical complications, n (%)	6 (28.6%)	2 (50%)	0.34	2 (11.1%)	6 (75%)	0.003
Pulmonary embolism	0	0		0	2 (25%)	
Deep vein thrombosis	1 (4.8%)	0		0	0	
Pulmonary infection	1 (4.8%)	1 (25%)		0	2 (25%)	
Cardiac distress	0	0		0	1 (12.5%)	
Cicatrization disorder	1 (4.8%)	0		0	0	
Urinary tract infection	1 (4.8%)	0		2 (11.1%)	0	
Nerve injuries	1 (4.8%)	1 (25%)		0	0	
Myocardial infarction	1 (4.8%)	0		0	0	
Haemorrhagic shock	0	0		0	1 (12.5%)	
Surgical site infection, *n* (%)	0	1 (16.7%)	0.24	2 (11.1%)	0	1.00
Revision, *n* (%)	3 (14.3%)	2 (50%)	0.23	2 (11.1%)	0	0.53
Secondary THA	3 (14.3%)	1 (16.7%)		0	0	
Surgical site infection	0	1 (16.7%)		2 (11.1%)	0	
Heterotopic ossification, *n* (%)	5 (23.8%)	2 (50%)	1.00	5 (27.8%)	1 (12.5%)	0.63

*ORIF* open reduction internal fixation, *DM-CHP* dual mobility-combined hip procedure,

*SA-ORIF* single approach ORIF, *CA-ORIF* combined approach ORIF, *SA-CHP* single approach DM-CHP,

*CA-CHP* combined approach DM-CHP, *THA* total hip arthroplasty

In the ORIF group, the anatomical reduction was observed in 72% (18/25 patients). The rate of post-operative secondary arthritis was 32% (9/25 patients) looking at all fracture patterns, with 87.5% in associated fracture patterns.

**Fig. 1:**
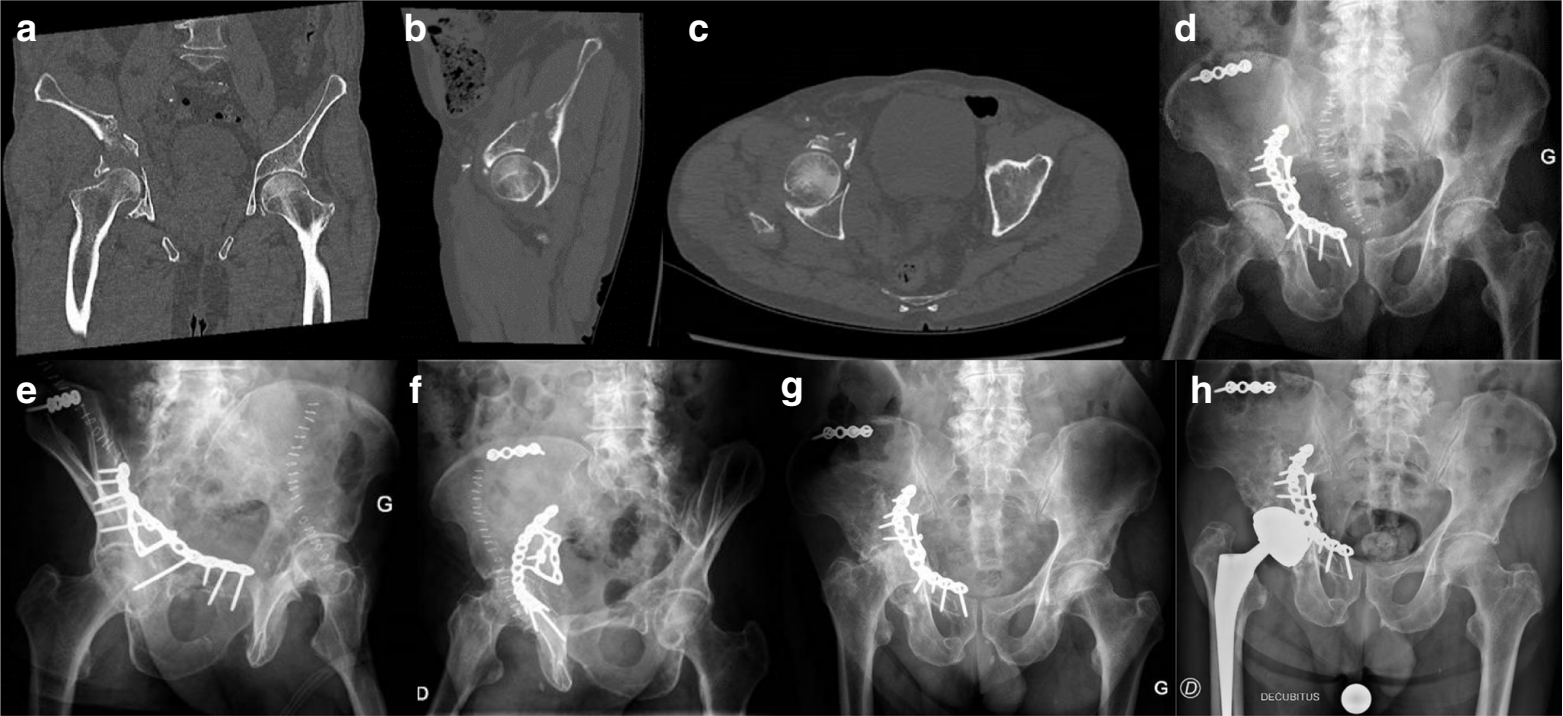
Pre-operative coronal, sagittal and axial views (**a**, **b** and **c**) on CT scan of an 87-year-old male patient who sustained an anterior column with posterior hemi-transverse fracture. (**d**, **e,** and **f**) Post-operative anteroposterior, obturator, and iliac X-rays after ORIF. (**g**) Anteroposterior pelvic X-ray at 15 months of follow-up showing osteonecrosis of the femoral head and acetabular collapse. (**h**) Anteroposterior pelvic X-ray after secondary THA with dual mobility cup.

The revision rate was 20% (5/25) in the ORIF group compared with 7.7% (2/26) in the DM-CHP group (Table 1). Reasons for revisions in ORIF were four secondary THA (three symptomatic osteoarthritis and one osteonecrosis) and one early infection. The four fracture patterns that needed secondary THA were as follow: one comminuted posterior wall fracture, two T-type fractures with severe dome comminution, and one anterior column with posterior hemi-transverse (AC-PHT) fracture (Fig. 1). The median time between ORIF and secondary THA was 11 months (range 6–24). Reasons for revisions in DM-CHP were two early infections. Two dislocations occurred: one early (2 weeks post-operative) and one delayed (1-year post-operative) due to a fall. Both unique dislocations were managed successfully by closed reduction.

### Subgroups analysis

**Fig. 2:**
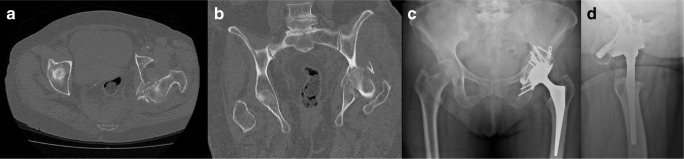
Pre-operative axial (**a**) and coronal (**b**) views on CT scan of an 82-year-old female patient who sustained a comminuted posterior wall fracture associated with posterior hip dislocation treated by DM-CHP with posterior approach only (SA-CHP). Post-operative anteroposterior pelvic (**c**) and axial hip (**d**) X-rays at 12 months of follow-up post-SA-CHP.

**Fig. 3:**
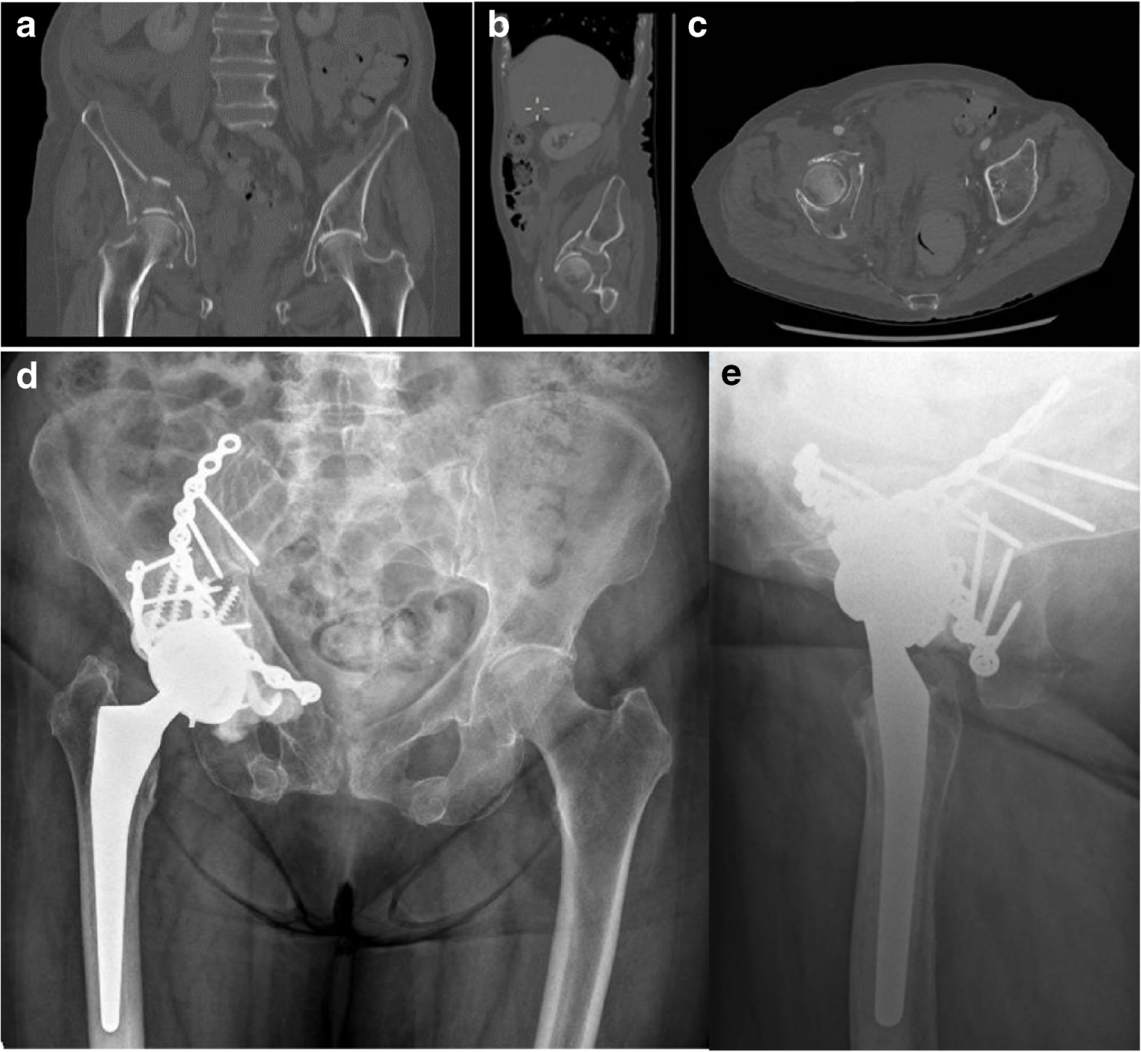
Pre-operative coronal, sagittal and axial views (**a**, **b,** and **c**) on CT scan of an 87-year-old female patient who sustained a both column fracture treated by DM-CHP with combined approach (CA-CHP). Anteroposterior pelvic (**d**) and axial hip (**e**) X-rays at 24 months of follow-up after CA-CHP showing implants in good position.

There were 21 patients in the SA-ORIF group and four patients in the CA-ORIF group. There were 18 patients in the SA-CHP group (Fig. 2) and eight patients in the CA-CHP group (Fig. 3). Mean surgery time and intra-operative blood loss were higher for combined approaches in each group. Early complication rate was 11.1% (2/18) in SA-CHP compared with 75% (6/8) in CA-CHP (*p* = 0.003). Early complication rate was 28.6% (6/21) in SA-ORIF compared with 50% (2/4) in CA-ORIF (*p* = 0.34) (Table 2).

The COR reconstruction measurements for DM-CHP are provided in Table 3. There was no statistically correlation between HHS and COR reconstruction.

**Table 3:** Biomechanical reconstruction of the centre of rotation and correlation with HHS

Parameter of reconstruction	Difference from the contralateral hip	P value	PCC
	(mm), median (range)		
Horizontal hip COR	+ 1.25 (− 11.1 to + 11.3)	0.26	0.25
Vertical hip COR	−2.4 (−36.1 to + 6.4)	0.43	− 0.18
Horizontal femoral offset	−2.1 (− 22 to + 17.6)	0.36	− 0.2
Limb discrepancy	+ 4.75 (− 8.3 to + 42)	0.29	0.24

*COR* center of rotation, *HHS* Harris Hip Score, *PCC* Pearson correlation coefficient, *mm* millimeters

## Discussion

This study showed no difference in term of functional outcome (HHS), non-fatal complication rate, and infection rate between ORIF and DM-CHP, which is in accordance with the systematic review of Capone et al. [8]

Our results showed a significantly higher blood loss and higher surgery time for the DM-CHP group. The subgroup analysis also showed higher blood loss and longer surgery time for the combined approach in both groups. Moreover, there was a statistically correlation between medical complication rate and combined approaches in the DM-CHP group which can be explained by the higher blood loss and increased surgery time due to the double approach and patient repositioning during surgery. In our study, 56% of all fractures involved the anterior column, and stabilization of this part of the acetabulum could not be achieved using a posterior approach [15]. Inspired by Tidemark’s work on primary THA with Burch-Schneider antiprotrusion cage [22], Boelch et al. [10] have implanted nine primaries THA by single approach (Bauer or Kocher-Langenbeck). The stability of the posterior column was one of the essential factors of the surgical strategy. More recently, Lont et al. [14] had shown the importance of posterior column stabilization associated with the prevention of central migration using a posterior column plate and an antiprotrusion cage. Stabilization of the posterior column only in acute THA leads to reduced operation time, easier approach, and low post-operative complications [14]. Mean surgery time was lower [10, 14, 22], mean blood loss was lower [10] or similar [14, 22], and mean HHS was higher for Tidemark et al. [22] in comparison with our CA-CHP group. On the other hand, Rickman et al. [12] used combined approaches on 20 elderly patients with only two post-operative complications. The mean surgery time was 193 minutes [12] compared with 212 minutes in our CA-CHP group. Our study strengthens the practice of using only the posterior approach for CHP in the elderly who sustained acetabular fractures.

In the present study, the anatomical reduction was obtained in more than 70% of all the 25 cases, which probably explains the relatively low rate of conversion to secondary THA (16%) [8]. All of our secondary THA after ORIF presented predictors of poor outcomes, namely, one posterior wall comminution, and three supero-medial (quadrilateral plate) comminutions (2 T-type fractures and 1 AC-PHT fracture). Kreder et al. [6] published that 54% of early hip replacement were done after primary ORIF for complicated posterior wall. Fica et al. [23] observed that T-type fractures were associated with poor clinical outcome. On the other hand, Matta et al. [4] have found 77% of good or excellent clinical results for 31 T-type fractures. Quadrilateral plate comminution was associated with poor post-operative reduction and low HHS [24]. We reported a higher revision rate in the ORIF group than in the DM-CHP group essentially represented by secondary THA, which is in accordance with the results of others study [10, 14] and systematic review [8].

Dislocation was one of the most common complications in primary THA for acetabular fracture, together with infection and HO. Herscovici et al. [15] reported a 23% dislocation rate in 22 elderly patients, which lead to five revisions. On the other hand, Salama et al. [11] reported no cases of dislocation in their series of 18 patients. We found only one study which mentioned DMC as a primary implant for THA for acetabular fractures in the elderly [13], with a dislocation rate of 11% in a cohort of 27 patients. Our dislocation was 7.7% (2/26) with non-surgical management in both cases. The theoretical aim of DMC is to increase stability by allowing a large, effective head-to-neck ratio, and by creating two articulating surfaces [25]. There are some well-described THA instability risk factors related to patients, implants, and surgical techniques. COR and offset reconstruction are two of the major factors involved in THA stability. One study recorded the biomechanical reconstruction of their acute THA for acetabular fractures [10] and showed relevant variations in the COR reconstruction. This coincides with our results. Their dislocation rate was 22% [10]. Our low dislocation rate could also be partially explained by the use of DMC. Outcomes of DMC were evaluated, especially in femoral neck fracture, showing a reduced revision risk for DMC THA due to dislocation [18].

This study has some limitations. It is a retrospective study with a small number of patients. In addition, the pre-operative functional scores of the hip (HHS) were not assessed, which would probably yield a useful perspective on the post-operative HHS score. Moreover, median follow-up times were relatively low. Thus, results and conclusions do not explore mid-term and long-term outcomes.

## Conclusion

DM-CHP has a similar short-term functional outcome compared with ORIF. Early medical complications are significantly higher when DM-CHP was performed with combined approaches due to higher blood loss and longer surgical time. Our study supports the importance of posterior approach only in acute THA for acetabular fracture in the elderly. Furthermore, this procedure leads to less revision than ORIF. To our knowledge, this is the first study which evaluates the potential of DMC in primary THA for acetabular fractures. DMC should have a place in the therapeutic option for acetabular fractures in high dislocation risk populations like elderly patients. DM-CHP could be a good therapeutic option in selected patients who are at risk of poor outcomes with ORIF and in case of higher instability risk. Further investigations and longer follow-up are necessary to confirm this conclusion.
